# Supplementary material for: A Comprehensive Review of Immunotherapy Clinical Trials for Metastatic Urothelial Carcinoma: Immune Checkpoint Inhibitors Alone or in Combination, Novel Antibodies, Cellular Therapies, and Vaccines
Source: Cancers (Basel). 2024 Jan 12;16(2):335. doi: 10.3390/cancers16020335 (PMC10813852; doi:10.3390/cancers16020335)
Supplement: Supplementary file 1 [file cancers-16-00335-s001.zip › cancers-2683397-supplementary.pdf]

Supplemental Materials

Supplemental Table S1. Clinical trials of bispecific antibodies for advanced solid tumors including metastatic urothelial carcinoma.

Regimen	Drug Classes	Disease/Patients	Trial	Phase	Status (March 2023)	Identifier	ORR
RO7247669 +/- tiragolumab vs atezolizumab	PD-1 x LAG-3 BsAb + anti-TIGIT vs anti-PDL1	1L platinum-ineligible mUC		2	Recruiting	NCT05645692	
Bintrafusp alfa + NHS-IL12 +/- SBRT	TGFβ x PDL1 BsAb + immuno-cytokine +/- radiation	Metastatic non-prostate GU cancers including mUC		1	Recruiting	NCT04235777	
RO7121661	PD-1 x TIM-3 BsAb	Advanced solid tumors		1	Active, not recruiting	NCT03708328	
LB1410	PD-1 x TIM-3 BsAb	Advanced solid tumors, lymphoma	Keyplus-001	1	Recruiting	NCT05357651	
EMB-02	PD-1 x LAG-3 BsAb	Advance solid tumors		1/2	Recruiting	NCT04618393	6.4% [1]
AK129	PD-1 x LAG-3 BsAb	Advanced solid tumors		1	Recruiting	NCT05645276	
INCA32459	PD-1 x LAG-3 BsAb	Advanced solid tumors, melanoma, HNSCC		1	Recruiting	NCT05577182	
AK112	PD-1 x VEGF BsAb	Advanced solid tumors		1	Recruiting	NCT04047290	23.5% [2]
AK112	PD-1 x VEGF BsAb	Advanced solid tumors		1/2	Active, not recruiting	NCT04597541	
AK112 + AK117	PD-1 x VEGF BsAb + anti-CD47	Advanced solid tumors w HNSCC dose expansion		1b/2	Recruiting	NCT05229497	
AK112 + AK117 +/- chemo	PD-1 x VEGF BsAb + anti-CD47 +/- chemo	Advanced solid tumors w HNSCC dose expansion		1b/2	Recruiting	NCT05214482	
XmAb®20717	PD-1 x CTLA-4 BsAb	Advanced solid tumors	DUET-2	1	Completed	NCT03517488	13.0% [3]
AK104	PD-1 x CTLA-4 BsAb	Advanced solid tumors		1/2	Completed	NCT04172454	
AK104 + AK117	PD-1 x CTLA-4 BsAb + anti-CD47	Advanced solid tumors w esophageal dose expansion		1/2	Recruiting	NCT05235542	
AK104 + AK119	PD-1 x CTLA-4 BsAb + anti-CD73	Advanced solid tumors		1/2	Recruiting	NCT05559541	
KN046 + MAX-40279	PD-L1 x CTLA-4 BsAb + multi-TKI	Advanced solid tumors		1/2	Not yet recruiting	NCT05425602	
B1962	PD-L1 x VEGF BsAb	Advanced solid tumors		1	Not yet recruiting	NCT05650385	

Y101D	PD-L1 x TGF-β BsAb	Advanced solid tumors		1	Unknown	NCT05028556	
QLS31901	PD-L1 x TGF-β	Advanced solid tumors		1	Unknown	NCT04954456	
TST005	PD-L1 x TGF-β	Advanced solid tumors		1	Terminated	NCT04958434	0 % [4]
ABL501	PD-L1 x LAG-3 BsAb	Advanced solid tumors		1	Recruiting	NCT05101109	
FS118	PDL1 x LAG-3 BsAb	Advanced solid tumors		1/2	Active, not recruiting	NCT03440437	2.3% [5]
HLX301	PDL1 x TIGIT BsAb	Advanced solid tumors, lymphoma		1/2	Recruiting	NCT05390528	
HLX301	PDL1 x TIGIT BsAb	Advanced solid tumors w a mUC dose expansion cohort D		1/2	Recruiting	NCT05102214	
SI-B003	PD-1 x CTLA4 BsAb	Advanced solid tumors		1	Recruiting	NCT04606472	16.1% [6]
XmAb®22841 +/- pembrolizumab	LAG3 x CTLA4 BsAb +/- anti-PD1	Advanced solid tumors	DUET-4	1	Completed	NCT03849469	
XmAb®23104 +/- ipilimumab	ICOS x PD1 BsAb +/- anti-CTLA4	Advanced solid tumors	DUET-3	1	Recruiting	NCT03752398	4.8% [7]
CDX-527	PD-L1 x CD27 BsAb	Advanced solid tumors		1	Completed	NCT04440943	[8]
IBI322	PD-L1 x CD47 BsAb	Advanced solid tumors, lymphoma		1	Completed	NCT04338659	
IBI322	PD-L1 x CD47 BsAb	Advanced solid tumors		1	Completed	NCT04912466 NCT04328831	20% [9]
IMM2520	PD-L1 x CD47 BsAb	Advanced solid tumors		1	Recruiting	NCT05780307	
BAT7104	PD-L1 x CD47 BsAb	Advanced solid tumors		1	Recruiting	NCT05767060, NCT05200013	
HX009	PD-1 x CD47 BsAb	Advanced solid tumors		2	Active, not recruiting	NCT04886271	
HX009	PD-1 x CD47 BsAb	Advanced solid tumors		1	Active, not recruiting	NCT05731752	
XmAb®808 +/- pembrolizumab	B7-H3 x CD28 BsAb +/- anti-PD1	Advanced solid tumors		1	Recruiting	NCT05585034	
INBRX-105 +/- pembrolizumab	PDL1 x CD137 BsAb +/- anti-PD1	Advanced solid tumors		1	Recruiting	NCT03809624	
ABL503	PDL1 x CD137	Advanced solid tumors		1	Recruiting	NCT04762641	
PRS-344	PD-L1 x CD137 BsAb anticalin fusion	Advanced solid tumors		1/2	Recruiting	NCT05159388	
MCLA-145	PDL1 x CD137 BsAb	Advanced solid tumors		1	Recruiting	NCT03922204	
FS222	PDL1 x CD137 BsAb	Advanced solid tumors		1	Recruiting	NCT04740424	
FS120 +/- pembrolizumab	OX40 x CD137 BsAb +/- anti-PD1	Advanced solid tumors		1	Recruiting	NCT04648202	
Cinrebafusp alfa (PRS-343)	HER2 x CD137 BsAb	HER2+ advanced solid tumors		1	Completed	NCT03330561	12% [10]

Cinrebafusp alfa (PRS-343) + atezolizumab	HER2 x CD137 BsAb + anti-PDL1	HER2+ advanced solid tumors		1b	Unknown	NCT03650348	
YH32367	HER2 x C137	HER2+ advanced solid tumors, breast + gastric dose expansion		1/2	Recruiting	NCT05523947	
M802	HER2 x CD3	HER2+ advanced solid tumors		1	Not yet recruiting	NCT04501770	
KM257	HER2 x HER2 BsAb	HER2+ advanced solid tumors		1	Not yet recruiting	NCT05320874	
TQB2930	HER2 x HER2 BsAb	Advanced solid tumors prioritizing HER2+		1	Recruiting	NCT05380882	
MBS301	HER2 x HER2 BsAb	HER2+ advanced solid tumors		1	Recruiting	NCT03842085	
IBI315	PD-1 x HER2 BsAb	HER2+ advanced solid tumors		1	Completed	NCT04162327	
IMM2902	HER2 x CD47 BsAb	HER2+ advanced solid tumors		1	Recruiting	NCT05076591	
KN026 + KN046	HER2 x HER2 BsAb + PDL1 x CTLA4 BsAb	HER2+ advanced solid tumors		1	Active, not recruiting	NCT04040699	57.1% [11]
SI-B001	EGFR x HER3 BsAb	Advanced epithelial tumors		1	Recruiting	NCT04603287	
BNA035	EGFR x CD137 BsAb	Advanced solid tumors		1	Recruiting	NCT05150457	
HLX35	EGFR x CD137 BsAb	Advanced solid tumors		1	Active, not recruiting	NCT05360381	
HLX35 + HLX10	EGFR x CD137 BsAb + anti-PD1	Advanced solid tumors		1	Not yet recruiting	NCT05442996	
MCLA-129	EGFR x c-MET BsAb	Advanced solid tumors		1/2	Recruiting	NCT04930432	
EMB-01	EGFR x c-MET BsAb	Advanced solid tumors, NSCLC dose expansion		1/2	Recruiting	NCT03797391	5.3% [12]
REGN7075 + cemiplimab	EGFR x CD28 BsAb + anti-PD1	Advanced solid tumors	COMBINE-EGFR-1	1/2	Recruiting	NCT04626635	
BCA101 +/- pembrolizumab	EGFR x TGF-β BsAb +/- anti-PD1	EGFR-driven advanced solid tumors		1	Recruiting	NCT04429542	27-58% [13, 14]
M1231	EGFR x mucin-1 BsAb	Advanced solid tumors		1	Completed	NCT04695847	
TJ033721	CD137 x claudin 18.2 BsAb	Advanced solid tumors		1	Recruiting	NCT04900818	
PM1009	TIGIT x PVRIG BsAb	Advanced solid tumors		1	Recruiting	NCT05607563	
GI-101 +/- pembrolizumab or lenvatinib or RT	CD80 x IL2R BsAb +/- anti-PD1 or TKI or radiation	Advanced solid tumors	KEYNOTE-B59	1/2	Recruiting	NCT04977453	12% [15]
ATOR-1015	CTLA-4 x OX-40 BsAb	Advanced solid tumors		1	Completed	NCT03782467	

EMB-09	PD-L1 x OX-40	Advanced solid tumors		1	Recruiting	NCT05263180	
GEN1044 (DuoBody®-CD3x5T4)	CD3 x 5T4 BsAb	Advanced solid tumors		1/2	Terminated	NCT04424641	2.7%
Orlotamab (MGD009)	CD3 x B7-H3 BsAb	B7-H3 expressing advanced solid tumors		1	Terminated	NCT02628535	
Obrindatamab (MGD009) + retifanlimab	CD3 x B7-H3 BsAb + anti-PD1	B7-H3 expressing advanced solid tumors		1	Completed	NCT03406949	
EMB-07	CD3 x ROR1 BsAb	Advanced solid tumors		1	Recruiting	NCT05607498	
ERY974	CD3 x glypican3 BsAb	GPC3+ Advanced solid tumors		1	Completed	NCT02748837	
JCJ-78306358	CD3 x HLA-G tumor Ab BsAb	Advanced solid tumors		1	Completed	NCT04991740	
GEM3PSCA	PSCA targeted T cell engager BsAb	Metastatic UC, RCC, prostate or NSCLC		1	Terminated	NCT03927573	

**Supplemental Table S2. Clinical trials of cellular therapies in advanced solid tumors including metastatic urothelial carcinoma.**						
**Regimen**	**Drug Classes**	**Target**	**Disease / Patients**	**Phase**	**Status** (March 2023)	**Identifier**
HBI 0201-ESO TCRT	TCR gene engineered lymphocytes	NY-ESO-1	NY-ESO-1 expressing metastatic cancers	1/2	Recruiting	NCT05296564
TAEST16001	TCR gene engineered lymphocytes	NY-ESO-1	Advanced solid tumors (not NSCLC) with HLA-A*0201 and ≥25% NY-ESO-1 cells by IHC	1	Completed	NCT03159585
FH-MagIC TCR-T cells + atezolizumab	TCR gene engineered lymphocytes + anti-PDL1	MAGE-A1	Metastatic MAGE-A1 expressing UC, TNBC or NSCLC. HLA-A*02:01.	1/2	Terminated (slow accrual)	NCT04639245
Anti-MAGE-A3 LHA-A*01 Restricted TCR-gene engineered lymphocytes	TCR gene engineered lymphocytes	MAGE-A3	Metastatic MAGE-A3 expressing UC, RCC, melanoma, cervical cancer, or breast cancer. HLA-A*01.	1/2	Terminated (slow accrual)	NCT02153905
CCT301-59	CAR-T cells	ROR2	ROR2 positive advanced solid tumors	1	Active, not recruiting	NCT03960060
Irradiated allogeneic donor lymphocyte infusion	Allogeneic donor lymphocytes		Advanced solid and hematologic malignancies. HLA partially matched related donors.	1	Completed	NCT00161187
SNK01 +/- trastuzumab or cetuximab	Autologous NK cells +/- anti-HER2 or anti-EGFR		HER2 or EGFR expressing advanced cancers	1/2	Withdrawn	NCT04464967
HER2 specific CAR-T cells + intratumoral CAdVEC	HER2- specific autologous CAR VST + intratumoral oncolytic adenovirus	HER2	HER2 positive advanced solid tumors	1	Recruiting	NCT03740256

**Supplemental Table S3. Clinical trials of vaccines and oncolytic viruses in advanced solid tumors including metastatic urothelial carcinoma.**						
**Regimen**	**Drug Classes**	**Target**	**Disease / Patients**	**Phase**	**Status** (March 2023)	**Identifier**
DC205-NY-ESO-1 +/- sirolimus	Intranodal dendritic cell vaccine +/- mTOR inhibitor	NY-ESO-1	NY-ESO-1 positive advanced solid tumors	1	Completed	NCT01522820
Personalized tumor neoantigen peptide vaccine + pembrolizumab	Personalized tumor neoantigen vaccine + anti-PD1		Advanced solid tumors	1	Recruiting	NCT05269381
DSP-7888 + ICI	WT1 immunotherapeutic peptide vaccine + anti-PD1	WT1	Advanced solid tumors	1b/2	Completed	NCT03311334
ADXS-NEO +/- pembrolizumab	Listeria monocytogenes-based patient-specific tumor neoantigen vaccine +/- anti-PD1		Advanced solid tumors	1	Terminated	NCT03265080
CDX-1307	hCG-β dendritic cell targeted vaccine		Advanced solid tumors	1	Completed	NCT00648102
CAdVEC + HER2 specific CAR-T cells	Oncolytic adenovirus + HER2 autologous CAR-T	HER2	Advanced solid tumors expressing HER2	1	Recruiting	NCT03740256
Enadenotucirev	Oncolytic chimeric adenovirus		Advanced solid epithelial tumors, metastatic colorectal, mUC	1/2	Completed	NCT02028442
CVA21 +/- pembrolizumab	Coxsackievirus A21 (V937) +/- anti-PD1	ICAM-1, DAF	NSCLC, mUC, CRPC, melanoma	1	Completed	NCT02043665
PF-07263689 (WO-12) +/- sasanlimab	Oncolytic vaccinia virus +/- anti-PD1		Advanced solid tumors	1	Terminated (business decision)	NCT05061537
YSCH-01	Recombinant L-IFN expressing oncolytic adenovirus		Advanced solid tumors	1	Recruiting	NCT05180851

Supplement References

1. Day, D., et al., *1028P Preliminary phase I results from a first-in-human study of EMB-02, a PD-1xLAG-3 bispecific antibody, in patients (pts) with advanced solid tumors.* Annals of Oncology, 2023. **34**: p. S625.
2. Coward, J., et al., *Safety and efficacy of AK112, an anti-PD-1/VEGF-A bispecific antibody, in patients with advanced solid tumors in a phase I dose escalation study.* Journal of Clinical Oncology, 2021. **39**(15_suppl): p. 2515-2515.
3. Shum, E., et al., *523 Preliminary clinical experience with XmAb20717, a PD-1 x CTLA-4 bispecific antibody, in patients with advanced solid tumors.* Journal for ImmunoTherapy of Cancer, 2021. **9**(Suppl 2): p. A553-A553.
4. Tolcher, A.W., et al., *A phase 1, first-in-human, open-label, dose escalation and dose expansion study of TST005 in patients with locally advanced or metastatic solid tumors.* Journal of Clinical Oncology, 2023. **41**(16_suppl): p. 2531-2531.
5. Yap, T.A., et al., *A Phase 1 First-in-Human Study of FS118, a Tetravalent Bispecific Antibody Targeting LAG-3 and PD-L1 in Patients with Advanced Cancer and PD-L1 Resistance.* Clin Cancer Res, 2023. **29**(5): p. 888-898.
6. Wang, Y., et al., *SI-B003 (PD-1/CTLA-4) in patients with advanced solid tumors: A phase I study.* Journal of Clinical Oncology, 2023. **41**(16_suppl): p. e14668-e14668.
7. Akce, M., et al., *A phase 1 multiple-ascending dose study to evaluate the safety and tolerability of XmAb23104 (PD-1 x ICOS) in subjects with selected advanced solid tumors (DUET-3).* Journal of Clinical Oncology, 2022. **40**(16_suppl): p. 2604-2604.
8. Sanborn, R.E., et al., *A phase 1 dose-escalation study of a PD-L1xCD27 bispecific antibody CDX-527 in patients with advanced malignancies.* Journal of Clinical Oncology, 2021. **39**(15_suppl): p. 2585-2585.
9. Wang, J., et al., *Abstract CT513: Phase I study of IBI322 (anti-CD47/PD-L1 bispecific antibody) monotherapy therapy in patients with advanced solid tumors in China.* Cancer Research, 2022. **82**(12_Supplement): p. CT513-CT513.
10. Ku, G., et al., *525O A phase I dose escalation study of PRS-343, a HER2/4-1BB bispecific molecule, in patients with HER2-positive malignancies.* Annals of Oncology, 2020. **31**: p. S462-S463.
11. Gong, J., et al., *339 Preliminary safety, tolerability and efficacy results of KN026 (a HER2-targeted Bispecific Antibody) in combination with KN046 (an anti-PD-L1/CTLA-4 Bispecific Antibody) in patients (pts) with HER2 aberrated solid tumors.* Journal for ImmunoTherapy of Cancer, 2020. **8**(Suppl 3): p. A207-A207.
12. Qing, Z., et al., *22P EMB-01: An EGFR-cMET bispecific antibody, in advanced/metastatic solid tumors phase I results.* Annals of Oncology, 2022. **33**: p. S39-S40.
13. Bedard, P.L., et al., *A phase 1 trial of the bifunctional EGFR/TGFβ fusion protein BCA101 alone and in combination with pembrolizumab in patients with advanced solid tumors.* Journal of Clinical Oncology, 2022. **40**(16_suppl): p. 2513-2513.
14. Hanna, G.J., et al., *Dose expansion results of the bifunctional EGFR/TGFβ inhibitor BCA101 with pembrolizumab in patients with recurrent, metastatic head and neck squamous cell carcinoma.* Journal of Clinical Oncology, 2023. **41**(16_suppl): p. 6005-6005.
15. Cho, B.C., et al., *718 Preliminary results of GII-101-P101 (Keynote-B59): GI-101 (CD80-IgG4 Fc-IL2v) as a single agent and in combination with pembrolizumab in patients with advanced and/or metastatic solid tumors.* Journal for ImmunoTherapy of Cancer, 2022. **10**(Suppl 2): p. A751-A751.
